# Supplementary material for: A genetic map of human metabolism across the allele frequency spectrum
Source: Nat Genet. 2025 Oct 3;57(10):2445–55. doi: 10.1038/s41588-025-02355-3 (PMC12513840; doi:10.1038/s41588-025-02355-3)
Supplement: Supplementary file 1 — Supplementary Note and Figs. 1–7. [file 41588_2025_2355_MOESM1_ESM.pdf]

nature genetics

Supplementary information https://doi.org/10.1038/s41588-025-02355-3

A genetic map of human metabolism across the allele frequency spectrum

In the format provided by the authors and unedited

Supplementary Note

Sex-differential effects at loci encoding metabolic genes

To assess whether our genetic analyses were driven by sex differences, we performed sex-stratified GWAS within the largest ancestry (EUR). We defined 'female' and 'male' sex including participants where the recorded sex and sex chromosomes aligned (XX for females and XY for males). The recorded sex was self-reported, and it was not possible to distinguish sex from gender. We acknowledge the importance of distinguishing between sex and gender in research and that chromosomal make-up does not always align with self-identified gender. In total, phenotypic, covariate and genetic data was fully available for 198,796 males and 235,850 females. GWASs were performed using REGENIE as described above. Per metabolic trait, we meta-analysed the sex-stratified results using the inverse-variance weighted model in METAL (v2020-05-05)[3] and clumped results on the heterogeneity p-value using plink (v2.00)[4] (--clump-p1 5 x 10^{-8}, --clump-r2 0, --clump-kb 2500). We considered loci putatively sex-differential where the heterogeneity p-value < 5 x 10^{-8}. We performed additional sensitivity analyses on the putatively sex-differential loci by assessing the influence of covariates confounded by sex (BMI, tobacco usage, alcohol consumption, lipid-lowering medication and diabetic medication). To properly model all gene by environment interactions[5], we fitted the following model per clump lead variant and associated metabolic phenotype, including the same set of covariates used in the original GWAS:

NMR phenotype ~ SNP + confounder + sex + age + fasting duration + PC1-10 + SNP*sex + SNP*confounder + sex*confounder + error

While we observed highly correlated effect sizes across female and male participants (median R^2: 0.98, range: 0.90 – 0.99), we also identified 360 putative sex-differential loci for 239 metabolic traits, representing 1,800 heterogenous associations in sex-stratified meta-analyses (heterogeneity p-value < 5 x 10^{-8}, see **Methods**). To rule out that sex-differential effects could be explained by other factors that differ between the sexes, we performed additional analyses identifying that sex-differential effects at one-third of loci (n=625, 34.7%) were attenuated when controlling for factors such as body mass index,

tobacco use, alcohol intake, and the use of lipid-lowering or diabetes medication (**Supplementary Fig. 2, Supplementary Table 4**). For loci unaffected by such additional factors, effect estimates were generally directionally concordant between the sexes but showed differences in magnitude (**Fig. 2a**). This is consistent with results previously observed for proteomics[6] and suggests that the majority of significant sex interactions do not reflect sex-discordant effects. We observed pleiotropic sex-differential loci associated with 30 or more NMR measures near established lipoprotein genes (*APOE, APOC1, LPL*) but also less established genes (*SIDT2*), where sex was the most likely modifying factor. These finding may help to better understand sex-specific cut-offs in cardiovascular risk assessment in clinical guidelines to initiate treatment with lipid lowering medication[7]. We found *CPS1* on 2q34 to show the strongest sex differences, in line with previous reports[8], with effect sizes for glycine being twice as large in females compared to males (rs1047891, beta females = 0.77, beta males = 0.34 s.d. units).

Refinement of regional associations through multi-ancestry fine-mapping

We next employed a two-stage strategy to refine regional associations to a small number of candidate causal variants. Firstly, we implemented fine-mapping in the largest group of European-ancestry participants. We then further refined the subset of loci with at least suggestive evidence across ancestries ($p<10^{-4}$) using trans-ancestral fine-mapping, leveraging the differential blocks of linkage disequilibrium (LD) despite vastly different sample sizes.

We first identified 3,007 statistically independent metabolite quantitative trait loci (mQTLs) associated with one or more NMR measure, representing a total of 43,322 credible set – NMR measurement pairs (**Supplementary Table 5**). This successfully defined 16,170 credible sets with a high-confidence variant (posterior inclusion probability (PIP) > 0.5). Among these were low-to-common frequent variants with functional consequences in metabolic genes, such as rs78734745 (MAF=0.8%; PIP=67.9%), a splice donor variant for *ME1*, associated with plasma citrate levels (beta=-0.11; p-value<1.6×10^{-21}). Lead fine-mapped mQTLs for a given NMR measure explained,

on average, 6.9% (range: 0.57% - 13.42%) of the variance in plasma concentrations (**Extended Data Fig. 4**).

Second, we aimed to use ancestrally diverse genetic data to refine the credible sets identified in the White European analyses. For credible sets that contained two or more variants in the European analyses, we checked for evidence of a genetic signal in the British-African and British-Central/South Asian ancestries ($P < 1.0 \times 10^{-4}$) at the same locus (±25kb on either side of the credible set). After these filters, we considered credible sets for fine-mapping across ancestries using MultiSuSiE[1]. We considered only quality-controlled variants that were prevalent in all populations (MAF>0.5%) and used the posterior inclusions probabilities from the European analyses as priors. LD matrices were calculated from a random subset of 50,000 White European participants for Europeans and using all available individuals for the British-African and British-Central/South Asian ancestries.

We thus leveraged the different LD-block structure among participants of British African and British Central South Asian ancestry to further refine credible sets that still contained >1 variant and for which the locus had at least suggestive evidence for significance in either ancestry ($P < 1.0 \times 10^{-4}$). Trans-ethnic fine-mapping led to an increase in the number of credible sets containing high-confidence variants (Europeans: 997, multi-ancestral: 1,794) and decreased the median credible set size from 9 to 4 variants, while increasing the median posterior inclusion probability from 0.06 to 0.16 (**Supplementary Fig. 3a-3b**). This included 1,107 (33.7%) credible sets with two or fewer variants, and 1,518 (45%) credible sets that were reduced in size by more than half. We note, however, that most eligible European credible sets were already comparatively small (median 9 variants), but sometimes still spanned multiple genes.

For example, a signal associated with mono-unsaturated fatty acids (MUFA) concentrations at 17q21.2 contained 76 genetic variants spread across several genes covering a 1Mb window in the European-only discovery. The signal was fine-mapped to as few as 4 variants (two intergenic, one <50kb distance to the gene body) after incorporating evidence from other ancestries (**Supplementary Fig. 3c**). Three of these

four variants mapped to the *PTRF/CAVIN1* gene, which plays a crucial role in the formation of caveolae that are particularly abundant in adipocytes. Thus, *PTRF/CAVIN1* has been linked to generalized lipodystrophies[2], providing a biologically plausible effector gene at this locus through trans-ancestral refinement of the credible set.

Phenotypic consequences of rare variation in metabolic genes

Rare inborn errors of metabolism are among the few disorders screened for at birth by most healthcare systems globally, as early intervention – such as appropriate substitution or dietary regimens – can prevent developmental issues and diseases later in life. We observed a more than 3-fold enrichment of genes previously linked to Mendelian diseases[9] ('OMIM genes') among those associated with NMR measures in gene burden and rare exonic variant analyses (odds ratio: 3.30; p-value<6.5×10^{-17}; **Supplementary Table 11**), in line with results reported from previous mGWAS[1,2,7,8]. For 15 out of 106 genes, we found evidence of significantly associated disease risk (p<7.5×10^{-7}), largely replicating signs and symptoms of corresponding rare disorders (**Supplementary Table 12**). Associations with NMR measures thereby represented different modes of action. For cardiovascular diseases, most prominently familial hypercholesterolemia (e.g., via *APOB*), they likely acted as mediators, whereas associations converging on *PKD1* for cystic kidney disease likely indicated disease consequences. We further observed less understood pleiotropic roles of OMIM genes. For example, rare predicted loss-of-function variants within *SMAD6* are known to cause, amongst others, malformations of bones, e.g., Craniosynostosis 7, characterised by malformations of the skull and subsequent brain damage, and we observed a strongly increased risk for other disorders of the cervical region (OR:28.8; 95%-CI: 10.3 – 80.5; p-value<1.4×10^{-10}), as well as significantly smaller VLDL particles (beta:-0.13; 95%-CI: -0.16 - -0.09; p-value< 1.5×10^{-9}) among rare variant carriers in UKB. The gene product, SMAD Family Member 6, suppresses TGF-beta signalling, which has known effects on bone morphogenetic proteins[14]. Independent evidence suggests that *SMAD6* downregulation reduces the expression of core genes involved in lipoprotein metabolism, such as *LDLR* [15], that may explain the disease-unrelated association.

When we tested more generally whether a rare variant burden in metabolic genes was associated with disease susceptibility, we observed a significant enrichment among susceptibility genes for endocrine and metabolic disorders, such as type 2 diabetes and different lipidemias but not among other disease categories (**Supplementary Fig. 5**).

Disease-wide Mendelian randomization screen for non-lipoprotein measures

We performed a phenome-wide Mendelian Randomisation (MR) screen using outcome summary statistics from the independent FinnGenn[16] cohort, release 11 (June 2024). We assessed 1394 outcomes with genome-wide significant signals ($p<5x10^{-8}$). We selected 21 non-lipid NMR biomarkers as exposure variables and assessed separately four sets of instruments as described previously for the cardiovascular Mendelian Randomisation analyses. We included two well-characterized lipid biomarkers (LDL-C and ApoB) as positive controls in the MR analyses. We performed MR using the TwoSampleMR package (v0.5.1), implementing the inverse-variance weighted and the MR-Egger methods. We discarded results with MR Egger p < 0.001, Cochran's Q p-value < 1.0×10^{-6} and results where the estimated effect was directionally discordant between the IVW and Egger methods.

We observed a strong decline, 29 to 13 metabolite – disease association with significant evidence (adjusted p-value < 0.05) from two-sample MR ('level effect') once subsetting to metabolite-specific instruments, indicating false-positive results due to pleiotropy (**Supplementary Table 17**).

We observed evidence for convergence of locus and level convergence for a risk-increasing effect of genetically predicted plasma glycoprotein acetyl concentrations on type 2 diabetes risk (odds ratio per 1 s.d. increase: 1.67; p-value$<3.9x10^{-7}$). The association persisted even after additional exclusion of variants with evidence for pleiotropy in the GWAS catalog (odds ratio: 1.69; p-value$<9.1x10^{-5}$). Notably, 'locus' convergence was based on the consistent effect of the rare loss-of-function variant chr20:44413714:C>T (MAF = 0.02%) within *HNF4A* on plasma glycoprotein acetyl concentrations (beta: 0.60; p-value$<8.3x10^{-15}$) and the cumulative effect of ultra-rare loss-of-function variants on type 2 diabetes risk (odds ratio: 2.68; p-value: $6.5x10^{-10}$).

However, we note that plasma glycoprotein acetyl concentrations proxy a complex chronic inflammatory state[17] that warrants further follow-up analysis to establish mechanistic links to type 2 diabetes. In contrast, previously reported associations between genetically predicted levels of branched-chain amino acids and type 2 diabetes reached at best nominal significance with a smaller effect size than previously estimated[18] (e.g., plasma leucine concentrations: odds ratio per s.d. unit: 1.19; p-value<0.02).

References

1. Rossen, J. *et al.* MultiSuSiE improves multi-ancestry fine-mapping in All of Us whole-genome sequencing data. *medRxiv* (2024) doi:10.1101/2024.05.13.24307291.
2. Adiyaman, S. C. *et al.* Congenital generalized lipodystrophy type 4 due to a novel PTRF/CAVIN1 pathogenic variant in a child: effects of metreleptin substitution. *J. Pediatr. Endocrinol. Metab. JPEM* **35**, 946–952 (2022).
3. Willer, C. J., Li, Y. & Abecasis, G. R. METAL: fast and efficient meta-analysis of genomewide association scans. *Bioinformatics* **26**, 2190–2191 (2010).
4. Chang, C. C. *et al.* Second-generation PLINK: rising to the challenge of larger and richer datasets. *GigaScience* **4**, 7 (2015).
5. Keller, M. C. Gene × environment interaction studies have not properly controlled for potential confounders: the problem and the (simple) solution. *Biol. Psychiatry* **75**, 18–24 (2014).
6. Koprulu, M. *et al.* Similar and different: systematic investigation of proteogenomic variation between sexes and its relevance for human diseases. *medRxiv* (2024) doi:10.1101/2024.02.16.24302936.

7. Michos, E. D., McEvoy, J. W. & Blumenthal, R. S. Lipid Management for the Prevention of Atherosclerotic Cardiovascular Disease. *N. Engl. J. Med.* **381**, 1557–1567 (2019).
8. Wittemans, L. B. L. *et al.* Assessing the causal association of glycine with risk of cardio-metabolic diseases. *Nat. Commun.* **10**, 1060 (2019).
9. Amberger, J. S., Bocchini, C. A., Schiettecatte, F., Scott, A. F. & Hamosh, A. OMIM.org: Online Mendelian Inheritance in Man (OMIM®), an online catalog of human genes and genetic disorders. *Nucleic Acids Res.* **43**, D789–D798 (2015).
10. Surendran, P. *et al.* Rare and common genetic determinants of metabolic individuality and their effects on human health. *Nat. Med.* **28**, 2321–2332 (2022).
11. Lotta, L. A. *et al.* A cross-platform approach identifies genetic regulators of human metabolism and health. *Nat. Genet.* **53**, 54–64 (2021).
12. Long, T. *et al.* Whole-genome sequencing identifies common-to-rare variants associated with human blood metabolites. *Nat. Genet.* **49**, 568–578 (2017).
13. Shin, S.-Y. *et al.* An atlas of genetic influences on human blood metabolites. *Nat. Genet.* **46**, 543–550 (2014).
14. Horiki, M. *et al.* Smad6/Smurf1 overexpression in cartilage delays chondrocyte hypertrophy and causes dwarfism with osteopenia. *J. Cell Biol.* **165**, 433–445 (2004).
15. Zhang, F., Sodroski, C., Cha, H., Li, Q. & Liang, T. J. Infection of Hepatocytes With HCV Increases Cell Surface Levels of Heparan Sulfate Proteoglycans, Uptake of Cholesterol and Lipoprotein, and Virus Entry by Up-regulating SMAD6 and SMAD7. *Gastroenterology* **152**, 257-270.e7 (2017).
16. Kurki, M. I. *et al.* FinnGen provides genetic insights from a well-phenotyped isolated population. *Nature* **613**, 508–518 (2023).

17. Ritchie, S. C. *et al*. The Biomarker GlycA Is Associated with Chronic Inflammation and Predicts Long-Term Risk of Severe Infection. *Cell Syst.* **1**, 293–301 (2015).

18. Lotta, L. A. *et al.* Genetic Predisposition to an Impaired Metabolism of the Branched-Chain Amino Acids and Risk of Type 2 Diabetes: A Mendelian Randomisation Analysis. *PLoS Med.* **13**, e1002179 (2016).

Supplementary Figures

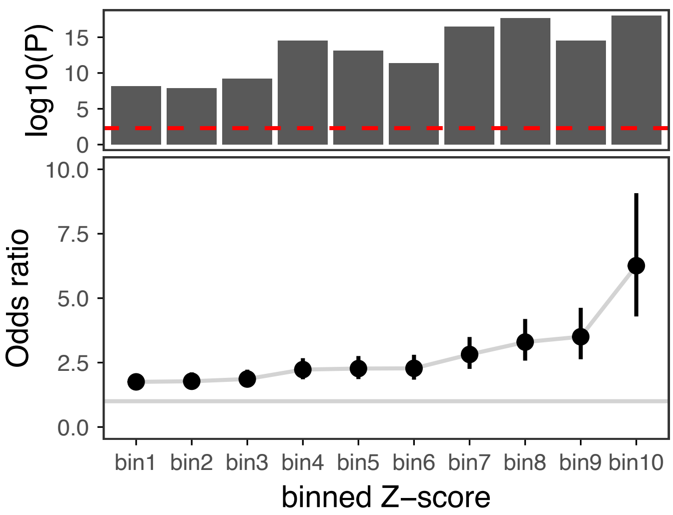

Supplementary figure 1. Enrichment of prioritised metabolic genes throughout bins of significance. Absolute Z-scores were binned in deciles and we extracted a list of genes proximal to fine-mapped lead variants. Enrichment of prioritised metabolic genes was calculated against the entire protein-coding background (n = 19747 genes). Odds ratio (dots) and standard errors (vertical lines) in the below plot and p-values in the top plot are derived from a two-sided Fisher's test. P-values are adjusted for multiple testing.

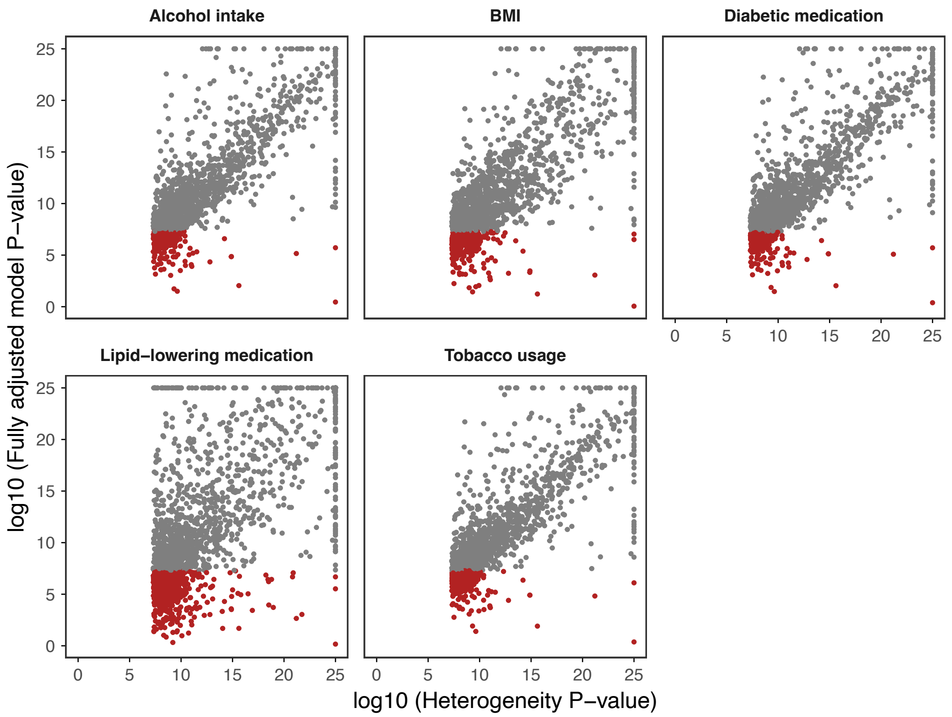

Supplementary figure 2. Comparison of P-values from 1800 putatively sex-differential loci identified through meta-analysis across the sexes. Shown are the METAL heterogeneity two-sided P-values (x-axis) and the raw two-sided p-values from the fully adjusted confounder model (y axis, see methods). Red dots represent loci where the sex-different effect was attenuated in the adjusted model and thus could be attributed to confounding effects.

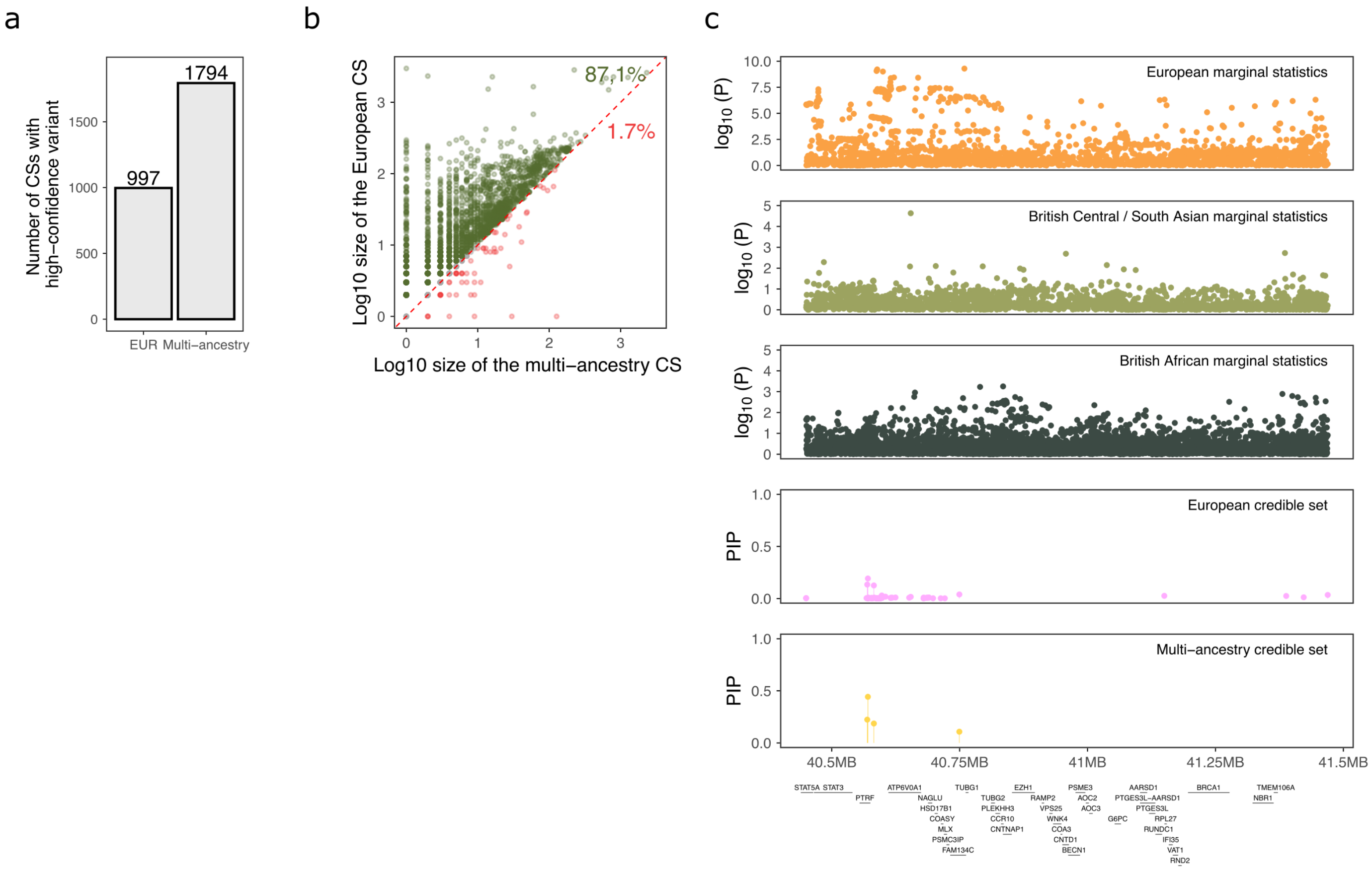

Supplementary Figure 3: Refinement of credible sets using multi-ancestral genetic data. A) Credible sets containing a high-confidence variant (posterior inclusion probability > 0.5) in the European-only and multi-ancestry fine-mapping analyses. **B)** Log-transformed size of European fine-mapped credible sets compared to multi-ancestral credible sets, shown for the credible sets that were considered in multi-ancestral finemapping analyses. The diagonal line represents equal credible set size between Europeans and multi-ancestral analyses, points above the diagonal indicate refined credible sets in multi-ancestral analyses. Percentages indicate the proportion of assessed credible sets that reduced in size through trans-ancestral finemapping (green) or increased (red). **C)** Example of a credible set before and after multi-ancestry fine-mapping, leading to assignment of a biologically plausible effector gene *PTRF*. P-values are two-sided, raw $-\log_{10}$(p-values) derived within each ancestral group separately (European n = 434,646, British-African n = 6,573, British-Central South Asians n= 8,796).

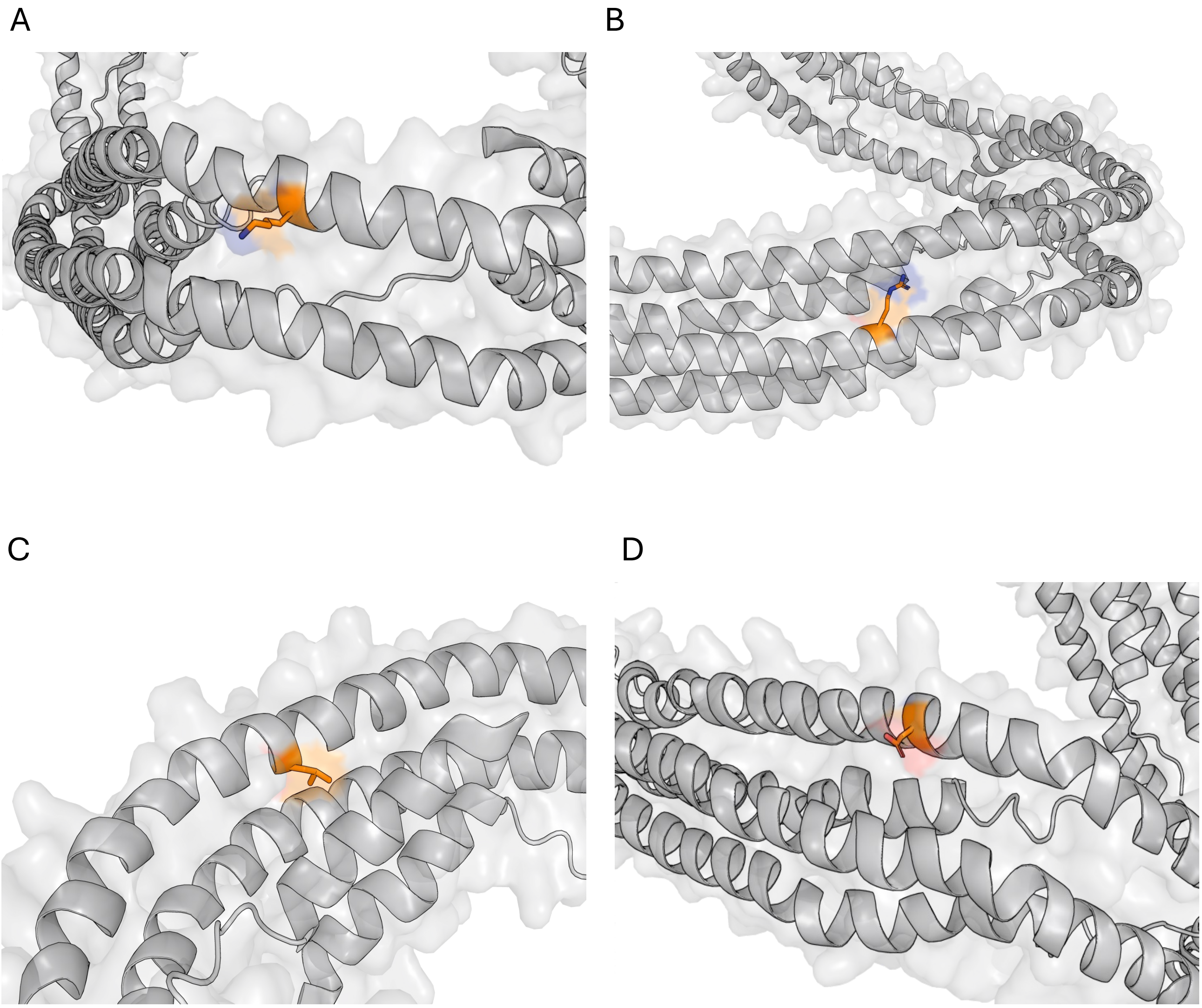

Supplementary figure 4. Location of 4 coding variants in APOA1 associated with NMR measures (PDB-ID 1AV1) coloured in orange; (**A**) p.Lys131del (**B**) p.Arg201Ser (**C**) p.Leu158Pro (**D**) p.Asp113Glu

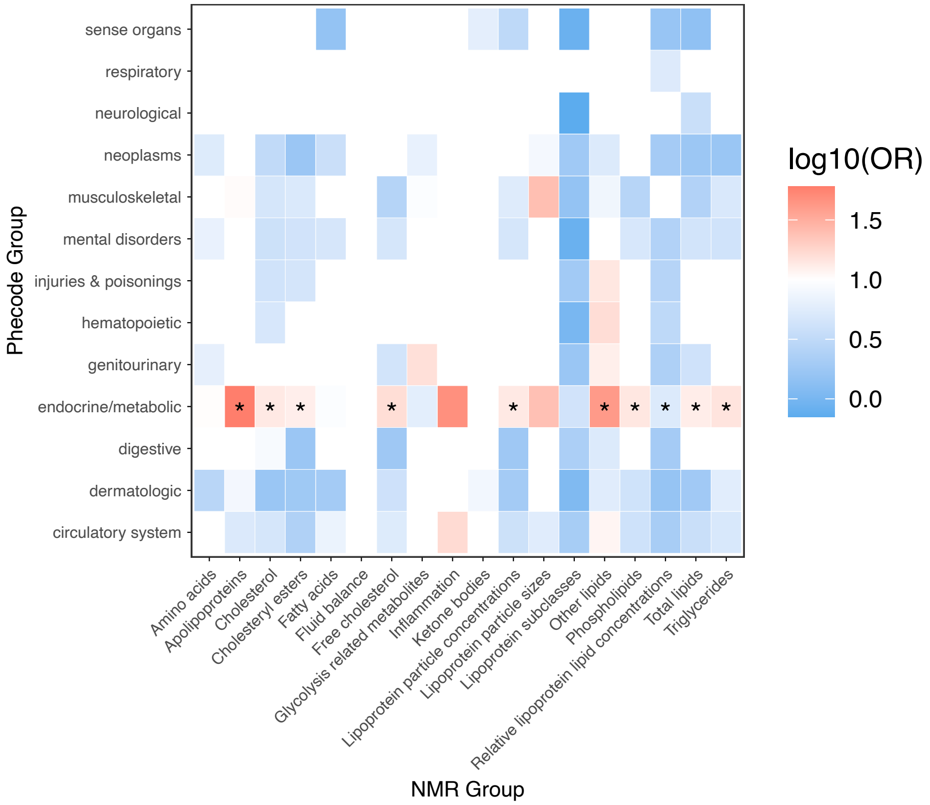

Supplementary figure 5: Enrichment of metabolite effector genes identified in whole-exome sequencing data amonst disease-related genes. Odds ratios are taken from a two-sided Fisher's test.

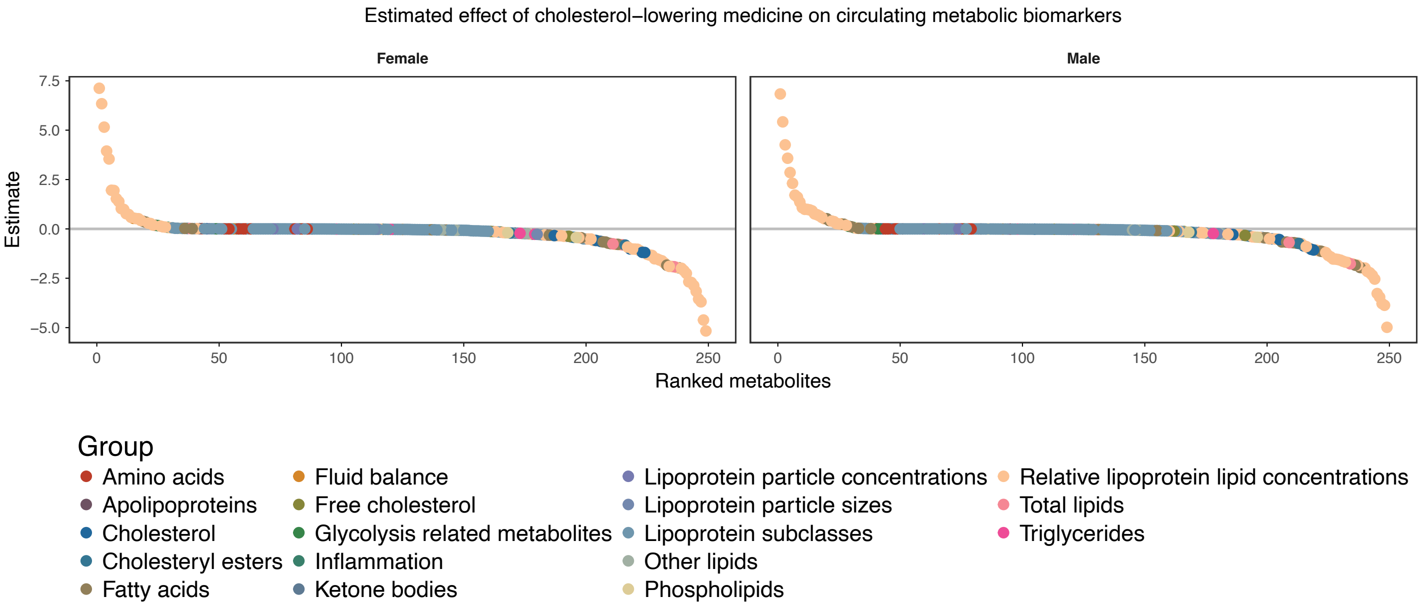

Supplementary figure 6: Effects of lipid-lowering medication on circulating metabolites. Each dot indicates one of 249 metabolites, ranked by the estimated effect of lipid-lowering medication on this metabolite. Metabolites are colored per biochemical class.

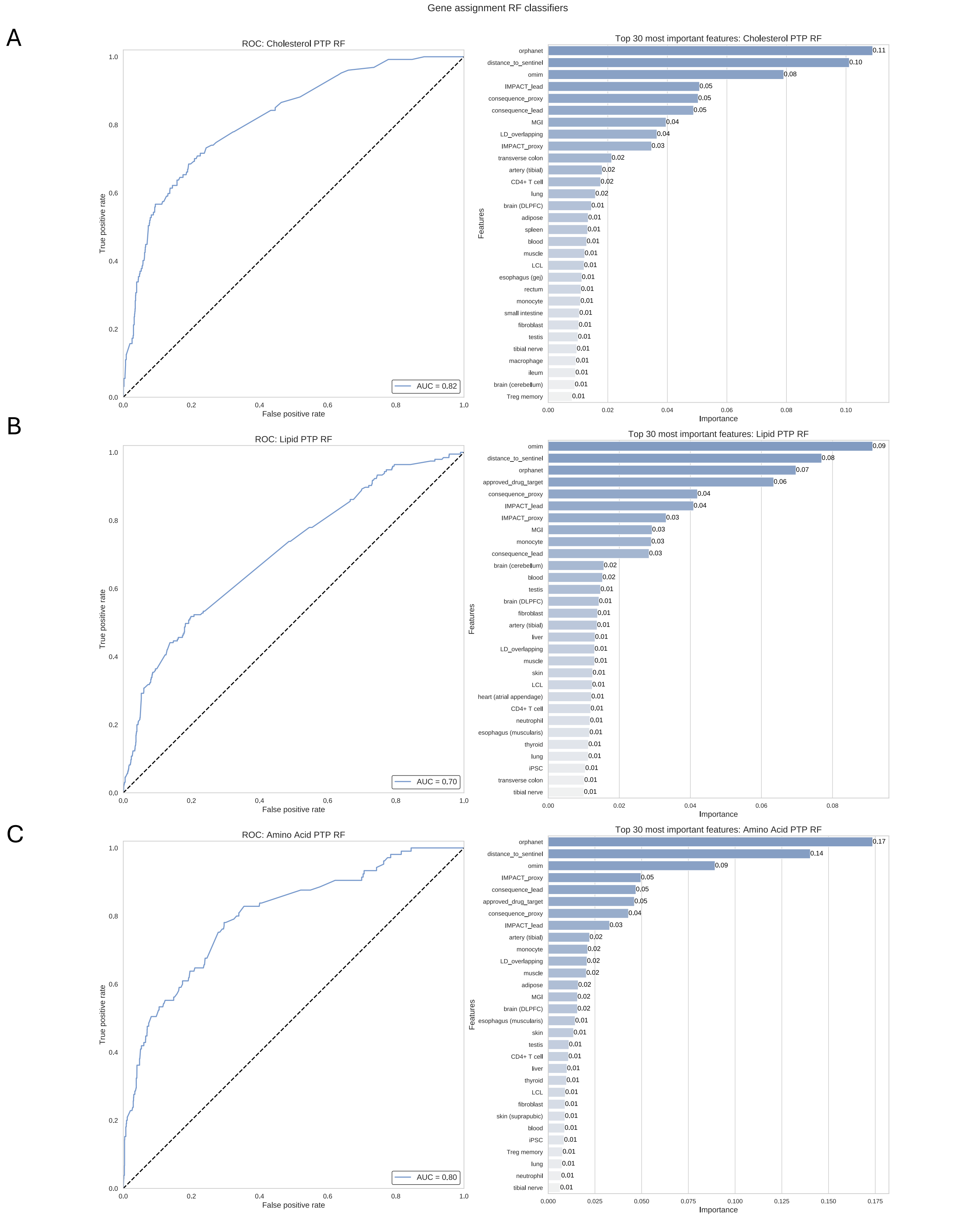

Supplementary figure 7. Area under the curve (left) and feature importance of the most predictive features (right) for models trained using a true positive set of genes annotated for cholesterol metabolism (**A**), lipid-related metabolism (**B**) and amino acid metabolism (**C**).
