# Supplementary material for: A genetic map of human metabolism across the allele frequency spectrum
Source: Nat Genet. 2025 Oct 3;57(10):2445–55. doi: 10.1038/s41588-025-02355-3 (PMC12513840; doi:10.1038/s41588-025-02355-3)
Supplement: Supplementary file 2 — Reporting Summary [file 41588_2025_2355_MOESM2_ESM.pdf]

nature portfolio

Corresponding author(s): Maik Pietzner & Claudia Langenberg

Last updated by author(s): 23 July 2025

Reporting Summary

Nature Portfolio wishes to improve the reproducibility of the work that we publish. This form provides structure for consistency and transparency in reporting. For further information on Nature Portfolio policies, see our Editorial Policies and the Editorial Policy Checklist.

Statistics

For all statistical analyses, confirm that the following items are present in the figure legend, table legend, main text, or Methods section.

n/a	Confirmed	
☐	☒	The exact sample size (*n*) for each experimental group/condition, given as a discrete number and unit of measurement
☐	☒	A statement on whether measurements were taken from distinct samples or whether the same sample was measured repeatedly
☐	☒	The statistical test(s) used AND whether they are one- or two-sided<br>*Only common tests should be described solely by name; describe more complex techniques in the Methods section.*
☐	☒	A description of all covariates tested
☐	☒	A description of any assumptions or corrections, such as tests of normality and adjustment for multiple comparisons
☐	☒	A full description of the statistical parameters including central tendency (e.g. means) or other basic estimates (e.g. regression coefficient) AND variation (e.g. standard deviation) or associated estimates of uncertainty (e.g. confidence intervals)
☐	☒	For null hypothesis testing, the test statistic (e.g. *F*, *t*, *r*) with confidence intervals, effect sizes, degrees of freedom and *P* value noted<br>*Give P values as exact values whenever suitable.*
☐	☒	For Bayesian analysis, information on the choice of priors and Markov chain Monte Carlo settings
☒	☐	For hierarchical and complex designs, identification of the appropriate level for tests and full reporting of outcomes
☐	☒	Estimates of effect sizes (e.g. Cohen's *d*, Pearson's *r*), indicating how they were calculated

Our web collection on statistics for biologists contains articles on many of the points above.

Software and code

Policy information about availability of computer code

Data collection	No software was used for data collection.
Data analysis	Data analyses were performed using REGENIE (v3.1.1), METAL (v2020-05-05), plink (v2), bcftools (v1.15.1), VEP (v106.1), REVEL, CADD (v1.6), LOFTEE, BEDtools (v2.3), LDStore v2. Further downstream analyses were implemented in R, using packages uknbnmr (v2.2), mice (v3.15), susieR (v0.12.35), igraph (v2.0.01), MungeSumStats (v1.13.2), coloc (v5.2.3), twoSampleMR (v0.5.1). Multi-ancestry finemapping was implemented in MultiSuSiE. Representative scripts used in analysis are freely available on Github (https://github.com/comp-med/ukb-mgwas)

For manuscripts utilizing custom algorithms or software that are central to the research but not yet described in published literature, software must be made available to editors and reviewers. We strongly encourage code deposition in a community repository (e.g. GitHub). See the Nature Portfolio guidelines for submitting code & software for further information.

Data

Policy information about availability of data

All manuscripts must include a data availability statement. This statement should provide the following information, where applicable:

- Accession codes, unique identifiers, or web links for publicly available datasets
- A description of any restrictions on data availability
- For clinical datasets or third party data, please ensure that the statement adheres to our policy

All individual-level data is publicly available to bona fide researchers from the UK Biobank (https://www.ukbiobank.ac.uk/). Full summary statistics for all analyses are publicly available through the NHGRI-EBI GWAS Catalogue (GWAS catalog identifiers GCST90497044 - GCST90501341, see Github repository)

Research involving human participants, their data, or biological material

Policy information about studies with human participants or human data. See also policy information about sex, gender (identity/presentation), and sexual orientation and race, ethnicity and racism.

Reporting on sex and gender	We defined 'female' and 'male' sex including participants where the recorded sex and sex chromosomes aligned (XX for females and XY for males). The recorded sex was self-reported, and it was not possible to distinguish sex from gender. We acknowledge the importance of distinguishing between sex and gender in research and that chromosomal make-up does not always align with self-identified gender. To assess whether our genetic analyses were driven by sex differences and whether our results were transferrable to both sexes, we performed sex-stratified GWAS within the largest ancestry (EUR).
Reporting on race, ethnicity, or other socially relevant groupings	We used previously published ancestral assignments by the pan-UKB consortium to assign individuals to ancestral groups, and made a further effort to .assign unclassified individuals to their respective ancestries based on a k-nearest neighbour approach using genetic principal components
Population characteristics	UK Biobank is a prospective cohort study from the UK that contains more than 500,000 volunteers between 40 and 69 years of age at inclusion. The cohort has been extensively described elsewhere (www.ukbiobank.ac.uk). Individuals were not directly selected for inclusion in the study on the basis of any disease or health parameter. The study consisted of 54.3% women and participants were on average 56.8 years old(s.d.:8.0).
Recruitment	All individuals between the age of 40-69 (men and women) who were registered with the National Health Service and living within a 25-mile radius from one of 22 recruitment centers spread across the United Kingdom were invited to participate in 2006-2010. Overall, about 9.2M individuals were invited to recruit around 0.5M individuals.
Ethics oversight	The UKBB was approved by the National Research Ethics Service Committee North West Multi-Centre Haydock.

Note that full information on the approval of the study protocol must also be provided in the manuscript.

Field-specific reporting

Please select the one below that is the best fit for your research. If you are not sure, read the appropriate sections before making your selection.

☒ Life sciences ☐ Behavioural & social sciences ☐ Ecological, evolutionary & environmental sciences

For a reference copy of the document with all sections, see nature.com/documents/nr-reporting-summary-flat.pdf

Life sciences study design

All studies must disclose on these points even when the disclosure is negative.

Sample size	We used the three major ancestral groups represented in the UK Biobank for our discovery analyses. The UKB (n≈500,000 individuals) is currently the largest available resource with linked genetic and metabolomics data.
Data exclusions	We excluded individuals where we did not have access to matching genotyping (array or sequencing-based) and metabolomics data. Furthermore, individual samples failing standard genotyping quality control or not assigned to one of the three major ancestral groups were excluded. These decisions were made before performing any statistical analysis.
Replication	We replicated our metabolome-wide genome-wide associations in the currently largest available meta-analysis of the same targeted metabolomics platform. Replication showed high concordance with the previously published studies.
Randomization	N/A - randomization occurred naturally as genetic variants were the exposure.
Blinding	N/A - genetic association testing does not require blinding as the effect of genetic variants on disease outcome is relative to the allele used as a reference.

Reporting for specific materials, systems and methods

We require information from authors about some types of materials, experimental systems and methods used in many studies. Here, indicate whether each material, system or method listed is relevant to your study. If you are not sure if a list item applies to your research, read the appropriate section before selecting a response.

Materials & experimental systems

n/a	Involved in the study	
☒	☐	Antibodies
☒	☐	Eukaryotic cell lines
☒	☐	Palaeontology and archaeology
☒	☐	Animals and other organisms
☒	☐	Clinical data
☒	☐	Dual use research of concern
☒	☐	Plants

Methods

n/a	Involved in the study	
☒	☐	ChIP-seq
☒	☐	Flow cytometry
☒	☐	MRI-based neuroimaging

Plants

Seed stocks	*Report on the source of all seed stocks or other plant material used. If applicable, state the seed stock centre and catalogue number. If plant specimens were collected from the field, describe the collection location, date and sampling procedures.*
Novel plant genotypes	*Describe the methods by which all novel plant genotypes were produced. This includes those generated by transgenic approaches, gene editing, chemical/radiation-based mutagenesis and hybridization. For transgenic lines, describe the transformation method, the number of independent lines analyzed and the generation upon which experiments were performed. For gene-edited lines, describe the editor used, the endogenous sequence targeted for editing, the targeting guide RNA sequence (if applicable) and how the editor was applied.*
Authentication	*Describe any authentication procedures for each seed stock used or novel genotype generated. Describe any experiments used to assess the effect of a mutation and, where applicable, how potential secondary effects (e.g. second site T-DNA insertions, mosiacism, off-target gene editing) were examined.*
